# Comparative study on the efficacy of peritoneal dialysis and hemodialysis in patients with end-stage diabetic nephropathy

**DOI:** 10.12669/pjms.36.7.2901

**Published:** 2020

**Authors:** Xiao-dong Xu, Xue Han, Yi Yang, Xu Li

**Affiliations:** 1 Xiao-dong Xu, Department of Medicine, Cangzhou Medical College, Hebei 061000, P. R. China; 2 Xue Han, Department of Medicine, Cangzhou Medical College, Hebei 061000, P. R. China; 3 Yi Yang, Department of Medicine, Cangzhou Medical College, Hebei 061000, P. R. China; 4 Xu Li, Dean’s Office, Cangzhou Medical College, Hebei 061000, P. R. China

**Keywords:** End-stage diabetic nephropathy, Hemodialysis, Peritoneal dialysis

## Abstract

**Objective:**

Diabetic nephropathy is a serious threat to human health, and its incidence is on the rise. End-stage diabetic nephropathy (ESDN) requires extra investigation due to its complexity and severity, as well as serious concurrent diseases. Our objective was to compare the efficacy of hemodialysis (HD) and peritoneal dialysis (PD) in the treatment of ESDN.

**Methods:**

Clinical data of 84 patients with ESDN admitted to our hospital from June 2016 to June 2018 were retrospectively analyzed. The patients were divided into an HD group that received hemodialysis and a PD group that received peritoneal dialysis. Their general conditions, biochemical indicators, residual renal function and incidence of complications were recorded and compared between the two groups.

**Results:**

(1) No significant difference in diastolic blood pressure, systolic blood pressure, body weight, or urine output was detected between the two groups at the beginning of dialysis (P>0.05). (2) Compared to the PD group, the HD group had significantly lower total cholesterol (TC) and triglyceride (TG) (P<0.05), and significantly higher total protein (TP) and albumin (ALB) after treatment (P<0.05). (3) The two groups also showed significant difference in residual renal function after treatment (P<0.05). (4) The HD group had significantly higher systolic pressure than the PD group after treatment (P<0.05). And more cases of infection were observed in the PD group than the HD group (P<0.05).

**Conclusion:**

Both HD and PD are used for treatment of ESDN, and can achieve similar calcium and phosphorus control. Compared to HD, PD has less adverse effect on hemodynamics and better preserves residual renal function, but is more likely to cause malnutrition and disorders of lipid metabolism. Therefore, choice of dialysis method should be based on specific conditions of each patient.

## INTRODUCTION

The incidence of diabetes is increasing year by year.^1^ Diabetic nephropathy is a common complication of diabetes, which seriously threatens human health.^2^ End-stage diabetic nephropathy (ESDN) is more troublesome and often complicated with serious comorbidities, making the treatment more difficult.^3^ Current treatment for ESDN mainly includes hemodialysis (HD), peritoneal dialysis (PD) and kidney transplantation.^4^ Although it has been shown that kidney transplantation has superior efficacy than PD and HD, the latter two remained the mainstream as only a small portion of patients had the chance to get kidney transplantation due to its high cost, limited kidney source, and requirements on patients’ physical conditions.^5^ With the advancement of dialysis technology, it is still controversial which renal replacement therapy is the best choice for DN patients.^6^

For patients with ESDN, PD has less adverse effect on hemodynamics and is less traumatic, but it may increase glucose absorption, cause obesity and lipid metabolism disorders and more protein loss, and consequently malnutrition. In addition, for patients on peritoneal dialysis, ultrafiltration volume should be carefully controlled, changes in peritoneal permeability and the occurrence of peritoneal fibrosis should be noticed. On the other hand, for patients on hemodialysis, establishment of vascular access may take great effort and the risk of low blood pressure during treatment should be carefully addressed. Therefore, choice of dialysis method for ESDN patients remains to be studied. In this study, clinical data of 84 cases of ESDN on dialysis from June 2016 to June 2018 were retrospectively analyzed. Clinical efficacy and commodities were compared between patients receiving hemodialysis and those receiving peritoneal dialysis, so as to make a better choice of treatment for patients with ESDN and improve their prognosis.

A total of 84 patients with ESDN admitted to our hospital from June 2016 to June 2018 were included in this study for retrospective analysis. The sample size was determined by calculations with the PASS software.

### Inclusion criteria:

1. ESDN patients with no history of hemodialysis or peritoneal dialysis before the study and aged 18 years or older.
2. Had maintenance dialysis (hemodialysis or peritoneal dialysis) for at least 3 years.

### Exclusion criteria:

1. Renal failure caused by acute kidney injury.
2. Not ESDN.
3. Receiving both hemodialysis and peritoneal dialysis.

### Ethical approval

This study was approved by the institutional ethics committee of XX Hospital (ethics number: 2016001), and written informed consent was obtained from all participants.

## METHODS

The patients were divided into an HD group that received hemodialysis and a PD group that received peritoneal dialysis. The HD group included 44 cases (26 males and 18 females); had an average age of 58.4 ± 6.3 years (46-75); and were on dialysis for an average of 12.79 ± 4.07 months. The PD group included 40 cases (24 males and 16 females); had an average age of 57.4 ± 7.7 years (48-74); and were on dialysis for an average of 12.74 ± 4.17 months The two groups of patients were comparable, with no significant difference in gender, age, course of disease and residual renal function before treatment (Table-I).

**Table-I:** General conditions of the two groups.

Variables	HD group	PD group
*Gender*		
Male	26	24
Female	18	16
Age (years)	58.4±6.3	57.4±7.7
Length of time on dialysis	12.79±4.07	12.74±4.17
GFR(ml/min/1.73m^2^)	8.23±0.95	8.21±0.97

### Treatment

Before dialysis, the patients were treated as needed to control blood glucose and blood pressure, and to correct anemia and calcium-phosphorus imbalance.

For hemodialysis, catheter to the central vein or arteriovenous fistula was used for vascular access, bicarbonate/reverse osmosis water solution was used as dialysate, and blood flow rate was set at 200-250 ml / minutes. And patients of the HD group received three hemodialyses a week, four hours each time. Patients in the PD group received peritoneal dialysis with the Y-type dialyzer (Baxter, U.S.) and dialysate containing 1.5%-4.25% sugar, 4 times a day, 2L each time.

### Indicators of interest

General conditions (including body weight, urine output and blood pressure), laboratory indicators (including blood urea nitrogen (BUN), creatinine (Scr), parathyroid hormone (PTH), blood calcium (Ca^2+^), blood phosphorus (P3+), hemoglobin (Hb), total protein (TP), albumin (ALB), triglyceride (TG), total cholesterol (TC), blood glucose, and residue renal function), as well as complications (including cardio-cerebrovascular diseases, bleeding, infection, malnutrition, arrhythmia, heart failure) of the two groups of patients were observed and recorded before and after dialysis.

### Statistical analysis

SPSS20.0 statistical software was used for data procession and analysis. Measurement data were represented as mean ± standard deviation (x ± s), and t test was used for comparison between the two groups; count data were represented as percentage (%), and compared with X^2^ test. P < 0.05 was considered statistically significant.

## RESULTS

Before dialysis, no significant difference in body weight, urine volume, diastolic blood pressure or systolic blood pressure was observed between the two groups(P> 0.05), but significant differences in urine volume and systolic blood pressure were observed between the two groups after dialysis. Table-II.

**Table-II:** Body weight, urine output and blood pressure of the two groups of patients

Variables	At the start of dialysis		After a year of dialysis	

	HD group	PD group	HD group	PD group
Body weight (kg)	60.2±5.9	62.6±6.1	58.2±5.8	60.2±5.7
Urine volume (ml)	935.5±133.6	1040.5±203.6	357.5±115.6	655.1±148.6*
Systolic blood pressure (mmHg)	161±15.2	158±15.7	156.3±14.8	143.1±14.9*
Diastolic blood pressure (mmHg)	85.3±8.4	82.1±9.6	79.5±7.8	77.4±8.1

* p<0.05 for comparison between the two groups after a year of dialysis.

After dialysis, the PD group had higher TC and TG levels (P<0.05), and lower TP and ALB levels than the HD group (P<0.05). Blood Ca^2+^ increased and P3+ decreased in both groups after treatment, but neither showed significant difference between the two groups at a year of dialysis. Table-III.

**Table-III:** Changes in the main laboratory indicators of the two groups.

Variables	HD group		PD group	

	At the start of dialysis	After a year of dialysis	At the start of dialysis	After a year of dialysis
Albumin (g/L)	27.1±2.39	31.9±2.14	27.3±2.57	28.7±1.91*
Total protein (g/L)	47.3±2.37	55.3±2.29	48.3±2.21	50.3±1.76*
Hemoglobin (g/L)	82.3±5.81	96.6±5.56	83.2±5.88	96.9±5.61
Total cholesterol (mmol/L)	5.91±0.22	5.96±0.19	5.92±0.21	6.36±0.29*
Triglyceride (mmol/L)	2.11±0.17	2.06±0.16	2.09±0.17	2.20±0.18*
Blood Ca^2+^ (mmol/L)	1.78±0.13	2.03±0.09	1.85±0.11	2.04±0.08
Blood P3+ (mmol/L)	2.05±0.12	1.89±0.09	2.01±0.11	1.84±0.10
Blood glucose (mmol/L)	9.95±1.23	7.66±0.83	9.36±2.09	7.8±0.87

* p<0.05 for comparison between the two groups after a year of dialysis.

### Changes of residual renal function of the two groups

After dialysis, Src, BUN, and PTH decreased in both groups, but showed no significant difference between the two groups (P>0.05). After a year of dialysis, the PD group had significantly higher GFR than the HD group (6.59±0.61 vs. 7.42±0.81 ml/min), indicating better preserve of residual renal function in the PD group. Table-IV.

**Table-IV:** Changes of residual renal function of the two groups.

Variables	HD group		PD group	

	At the start of dialysis	After a year of dialysis	At the start of dialysis	After a year of dialysis
Blood creatinine (umol/L)	781.5±172.9	621.6±86.1*	791.2±193.2	680.2±95.7
Blood urea nitrogen (mmol/L)	21.5±3.6	18.0±3.2*	21.5±3.9	18.1±3.7*
PTH (pg/ml)	261±87.2	209.1±65.7*	245.3±84.2	213.1±64.9*
GFR (ml/min/1.73m^2^)	8.23±0.95	6.59±0.61	8.21±0.97	7.42±0.81#

* p<0.05 for comparisons between data of the same group before and after a year of dialysis

# p<0.05 for comparisons between the two groups after a year of dialysis.

After a year of dialysis, more cases of concomitant hypertension were observed in the HD group than the PD group (P<0.05). But the PD group had significantly higher rate of infections than the HD group (mostly peritonitis, 14/40 vs. 3/44, P<0.05). Table-V.

**Table-V:** Incidence of adverse reactions of the two groups.

Adverse reactions	HD group (n = 44)		PD group (n = 40)	

	At the start of dialysis	After a year of dialysis	At the start of dialysis	After a year of dialysis
Hypertension	40	32	36	20*
Arrhythmia	6	8	6	4
Heart failure	10	6	8	4
Infection	10	3	12	14*

* p<0.05 for comparison between the two groups after a year of dialysis.

## DISCUSSION

Diabetic nephropathy is a serious complication of diabetes, and is characterized by high incidence and poor prognosis, representing an important cause of death in patients with diabetes.^7^ Diabetic nephropathy has become a major cause of end-stage renal disease. At the end stage of DN, patients often develop serious complications. Accurate and timely diagnosis, and prompt treatment is the key to satisfactory prognosis. Renal replacement therapies are currently the major treatment for ESDN. Both HD and PD can significantly extend survival of patients with ESDN, and a lot of studies have compared the efficacy and incidence of complications between HD and PD. In the early stages of dialysis, peritoneal dialysis can achieve better efficacy than hemodialysis, but hemodialysis tends to result in higher survival rate than peritoneal dialysis among ESDN patients. Hemodialysis can quickly remove toxins from the body, thus improve symptoms such as nausea and vomiting, reduce edema and improve appetite.^8^ While peritoneal dialysis is more continuous and stable, resulting in higher quality of life - intake of water and salt depends on daily clearance, which avoids water-sodium retention and maintains a relative balance of the body. For both groups, dialysis relieved the discomfort symptoms, improved appetite, reduced endotoxin levels, and corrected calcium and phosphorus metabolism disorders, so both HD and PD are effective treatment for patients with ESDN.^9^

It was observed in this study, urine volume significantly decreased in the HD group, but was largely maintained in the PD group, suggesting that PD may better preserve residual renal function than HD. PD also had less adverse effect on hemodynamics than HD, the possible causes may include no need to establish arteriovenous fistula; more stable PD and electrolyte balance; continuous and stable toxin removal; no significant changes in heart filling, myocardial contraction or oxygen demand during and between dialyses; better control of blood pressure; and better preserve of residual renal function. Hemodialysis has significant influence on circulatory dynamics, and use of anticoagulants during hemodialysis often leads to complications such as disequilibrium syndrome, and cardiovascular and cerebrovascular diseases.^10^,^11^ Blood pressure of the HD group was not well controlled, even after administration of antihypertensive drug for some patients. The causes may include water-sodium retention, insufficient ultrafiltration, increased vagal tone, and HD-related drug absorption and poor dialysis clearance.

Malnutrition and dyslipidemia are more commonly observed in patients on PD than those on HD.^12^,^13^ In this study, the lower total protein and albumin levels in the PD group appear to be related to higher level of protein loss. and the decreased cholesterol and triglyceride levels may be caused by increased glucose intake associated with PD, and increased synthesis of these lipids in the liver in response to hypoproteinemia. In recent years, the introduction of novel dialysates has somewhat reduced PD-related lipid metabolism and nutritional disorders. Some studies have shown that dialysate supplemented with amino acids and glucose at a certain ratio can provide protein and energy needed by the body, which is particularly useful for patients with insufficient food intake.^14^

The incidence of PD-related peritonitis also significantly decreased with technological advances.^15^ Infections were more common in the PD group than the HD group, which may be caused by weakened immunity due to significant protein loss.^16^ Therefore, patients on PD should pay more attention on diet, taking appropriate amount of high-quality protein and foods rich in vitamins and cellulose, and prevent diarrhea through food hygiene. In addition, peritoneal dialysis patients should be promptly followed up, with special attention on residual renal function as well as prevention and treatment of complications.^17^

At present, choices for the method and timing of dialysis are the major problems when treating patients with ESDN, and there are different views about them.^18^ For timing of dialysis, the US NKF-K/DOQI pointed out that dialysis for ESDM patients should be started when there is significant deficiency in renal function. It has been recommended that patients with SCR>442umol/L and CCR<15ml/min should receive dialysis, and for those with early occurrence of uremia, dialysis should be started even earlier, as soon as CCR reduced to below 20ml/minutes. Survival of ESDN patients on PD is closely related to residual renal function at the start of dialysis, nutritional status and comorbidities.^19^ So, timing of dialysis should be chosen based on comprehensive consideration of the patient’s kidney function, nutritional status and complications. In clinical practice, choice of dialysis method should also be made based on comprehensive evaluation of patient’s specific conditions, such as age, economic status, family environment and medical conditions, condition of the blood vessels, history of abdominal surgeries, general conditions, and major comorbidities before dialysis, etc. For patients with severe edema, hyperkalemia, or those who can’t have PH due to a history of abdominal surgeries, HD should be the choice. And for those who live in remote areas, have poor vascular conditions for establishment of vascular access, or have severe cardiovascular and cerebrovascular diseases, peritoneal dialysis should be the choice. Elderly patients with ESDN may choose PD at first to better preserve residual renal function, then switch to HD as needed.

Therefore, patients with ESDN should start dialysis as early as possible and chose the dialysis method most appropriate for each patient, which may change as the disease progresses. In addition, sufficient attention should be paid to control of blood sugar, blood lipids, and blood pressure, to improve nutritional status of patients, and to prevent various complications, so as to improve survival rate and quality of life of the patient.

### Limitations of the study

This study is limited by its retrospective nature: prospective randomized controlled studies should be performed in the future. And prognosis of ESDN patients on the two types of dialysis and relevant risk and protective factors should be further investigated.

## CONCLUSION

Both HD and PD are used for treatment of ESDN, and can achieve similar calcium and phosphorus control. HD showed better efficacy than PD in improving serum total protein and albumin. PD can better preserve residual renal function of patients with ESDN, but it tends to cause dyslipidemia. Patients with ESDN should start renal replacement therapy as early as possible, and the choice of dialysis method should be personalized and adjusted over time.

### Authors’ Contributions:

**XDX and** **XL** designed this study and prepared this manuscript, and are responsible and accountable for the accuracy or integrity of the work.

**XH** collected and analyzed clinical data

**YY** significantly revised this manuscript.
